# *Cardiac troponin T* is necessary for normal development in the embryonic chick heart

**DOI:** 10.1111/joa.12486

**Published:** 2016-05-19

**Authors:** Jennifer England, Kar Lai Pang, Matthew Parnall, Maria Isabel Haig, Siobhan Loughna

**Affiliations:** ^1^ School of Life SciencesMedical SchoolUniversity of NottinghamNottinghamUK

**Keywords:** atrial septal defects, cardiac troponin T, congenital cardiac diverticula, heart development, structural protein

## Abstract

The heart is the first functioning organ to develop during embryogenesis. The formation of the heart is a tightly regulated and complex process, and alterations to its development can result in congenital heart defects. Mutations in sarcomeric proteins, such as alpha myosin heavy chain and cardiac alpha actin, have now been associated with congenital heart defects in humans, often with atrial septal defects. However, cardiac troponin T (cTNT encoded by gene *TNNT2*) has not. Using gene‐specific antisense oligonucleotides, we have investigated the role of cTNT in chick cardiogenesis. *TNNT2* is expressed throughout heart development and in the postnatal heart. TNNT2‐morpholino treatment resulted in abnormal atrial septal growth and a reduction in the number of trabeculae in the developing primitive ventricular chamber. External analysis revealed the development of diverticula from the ventricular myocardial wall which showed no evidence of fibrosis and still retained a myocardial phenotype. Sarcomeric assembly appeared normal in these treated hearts. In humans, congenital ventricular diverticulum is a rare condition, which has not yet been genetically associated. However, abnormal haemodynamics is known to cause structural defects in the heart. Further, structural defects, including atrial septal defects and congenital diverticula, have previously been associated with conduction anomalies. Therefore, to provide mechanistic insights into the effect that cTNT knockdown has on the developing heart, quantitative PCR was performed to determine the expression of the shear stress responsive gene *NOS3* and the conduction gene *TBX3*. Both genes were differentially expressed compared to controls. Therefore, a reduction in cTNT in the developing heart results in abnormal atrial septal formation and aberrant ventricular morphogenesis. We hypothesize that alterations to the haemodynamics, indicated by differential *NOS3* expression, causes these abnormalities in growth in cTNT knockdown hearts. In addition, the muscular diverticula reported here suggest a novel role for mutations of structural sarcomeric proteins in the pathogenesis of congenital cardiac diverticula. From these studies, we suggest *TNNT2* is a gene worthy of screening for those with a congenital heart defect, particularly atrial septal defects and ventricular diverticula.

## Introduction

Congenital heart defects (CHDs) occur in approximately one in 180 live births (British Heart Foundation www.bhf.org.uk), and are a result of abnormal heart development. Cardiogenesis is initiated early in vertebrate development [Hamburger and Hamilton (HH) stage 10 (during day 2) in chick] (Hamburger & Hamilton, 1951). It begins by the formation of a simple, almost symmetrical heart tube which beats in a peristaltic motion. This process is made possible due to the assembly of a contractile unit, the sarcomere, which contains various structural proteins produced by the myocardial cell population, including myosin, actin and associated proteins tropomyosin and troponin (England & Loughna, 2013). As the heart tube matures, the primitive atrium becomes divided by the formation of a septum primum. This septum is initiated from the dorsocranial atrial wall at approximately HH14 (early day 3) and grows towards the developing endocardial cushions in the atrioventricular canal and is completed by HH24 (early day 5). Trabeculae appear in the primitive ventricle at HH17 and later contribute to ventricular septation, which is initiated by HH23 (day 4) and is completed by HH29–30 (day 7).

Although much progress has been made in the understanding of the complexity of cardiogenesis and its contribution to CHDs, gaps in our knowledge still remain. Several mutations in the genes of structural proteins have now been linked to CHDs such as *myosin heavy chain* (*MYH*) *6, MYH7* and *alpha cardiac actin* (Ching et al. 2005; Budde et al. 2007; Matsson et al. 2008). Mutations in all three of these structural proteins have been associated with one of the most common CHDs, atrial septal defects (ASDs), which occur in approximately one in 1500 live births. Congenital cardiac diverticulum, a localized protrusion of the endocardium and myocardium commonly associated with the left ventricular chamber of the heart that has normal contractility, is reported to occur at a frequency of approximately 0.04% in the general population (Marijon et al. 2006; Ohlow, 2006). In contrast to ASDs, to our knowledge, genetic mutations linked to congenital cardiac diverticula have not been reported.

Troponin T (TNT) is a 30–35 kDa protein that binds the troponin complex to tropomyosin and actin, serving as a scaffold and holding the thin filaments in contact. The N‐terminus of TnT binds to tropomyosin while the C‐terminus binds to the remaining troponin complex (Heeley et al. 1987). The human *TNNT2* gene, which encodes cardiac TNT (cTNT), is located on chromosome 1 and is alternatively spliced, leading to the creation of four different isoforms (Mesnard et al. 1995). The differential splicing for *TNNT2* is developmentally regulated, where isoforms 1, 2 and 4 are predominantly expressed in the human fetal heart, and isoform 3 (exon 5 absent) is expressed in the adult heart (Anderson et al. 1991). Thus, some isoforms are present only in specific stages of development. Interestingly, the fetal isoforms are re‐expressed at the mRNA and protein level in individuals with heart failure (Solaro et al. 1993; Anderson et al. 1995). Cardiac troponin T mutations have been associated with cardiomyopathies in humans and are inherited as autosomal dominant traits (Lu et al. 2013). Although mutations in *TNNT2* may result in mild hypertrophy, they can also cause sudden cardiac death (Watkins et al. 1995; Lu et al. 2013). Currently, over 100 mutations have been identified in troponin subunits associated with hypertrophic, dilated and restrictive cardiomyopathies, with over 50 of those found in cardiac troponin T (Lu et al. 2013). However, to date, *TNNT2* has not been linked to CHDs.

Targeted manipulation of *Tnnt2* prior to heart development in the mouse, axolotl and zebrafish has been studied. The homozygous null mouse (*Tnnt2* ^–/–^) was not viable beyond embryonic day (E)10.5 with no contractile activity observed, consistent with a lack of sarcomeric organisation (Ahmad et al. 2008). Disorganized myofibrils were also seen in the Mexican axolotl mutant heart (gene *c* mutant) (Fransen & Lemanski, 1989). Morpholino knockdown of cTnt in the zebrafish did not affect the formation of Z‐bodies, the initial structure for myofibrillogenesis; however, the Z‐bodies never assembled further into pre‐myofibrils (Huang et al. 2009). Thus, *Tnnt2* is thought to play a key role in myofibrillogenesis.

Here we confirm and describe the expression of *TNNT2* isoforms in the chick heart at different developmental ages. In this study, targeted knockdown of cTNT in the chick was performed at HH11, after the heart tube has formed. TNNT2‐morpholino (TNNT2‐MO) treatment at this stage led to the development of diverticula in the primitive ventricular wall. Furthermore, the size of the atrial septa and the number of ventricular trabeculae were reduced, although abnormalities in sarcomeric assembly and maturation were not seen *in vitro*. Quantitative PCR (qPCR) revealed that TNNT2‐MO treatment during cardiogenesis results in changes to the cardiac conduction transcription factor *TBX3*. In addition, the shear stress responsive gene *nitric oxide synthase 3* (*NOS3*) was differentially expressed in the TNNT2‐MO‐treated hearts. We provide evidence of novel roles for *TNNT2* in heart development, and suggest it as a candidate gene for CHDs, in particular ASDs and congenital cardiac diverticula.

## Methods

### Tissue collection

White fertile chicken eggs (*Gallus gallus*; Dekalb White strain; Henry Stewart, UK) were incubated at 38 °C in a humidified atmosphere with constant rotation prior to opening (Bellairs & Osmond, 2005). The Hamburger and Hamilton (HH) staging system was used to age developing chicks (Hamburger & Hamilton, 1992) and once the incubation time was complete, a small window was made in the egg. For RNA isolation, the hearts were immediately dissected and snap frozen in liquid nitrogen prior to storage at −80 °C. For TNNT2‐MO treatment, 3–5 mL of albumin was removed with a syringe to separate the embryo from overlying membranes. After treatment, the eggs were sealed with masking tape and placed back in the incubator until harvesting.

### RNA isolation and RT‐PCR

RNA was extracted from chick HH12, 14, 19, 22, 24, 26 and 34 hearts, neonatal (day 1) and adult atria and ventricles as previously described (Rutland et al. 2011). Reverse transcription reactions were performed using 1 μg RNA and random primers with SuperScript III Reverse Transcriptase (Invitrogen, UK) following manufacturer's instructions. A primer pair was designed to chick *TNNT2* in the region where alternate splicing occurs (exon 5), giving rise to both fetal and adult isoforms of *TNNT2* (forward 5′CGGACTCTGAAGAGGTCGTT3′ and reverse 5′TTGGGCTTTGATTCACCTTC3′). GoTaq^®^ polymerase was used (Promega, UK). The PCR reaction was carried out 58 °C and 72 °C for annealing and extension for 33 cycles, respectively. PCR products were resolved on a 2.5% agarose gel and were visualized under UV light using GeneGenius Bioimaging System (Syngene, UK). PCR products were sequenced as previously described (Rutland et al. 2011).

### Morpholino design and application

Two antisense oligonucleotides morpholinos were designed against the *TNNT2* ATG start site and to a non‐overlapping upstream region (ENSGALT00000000401; morpholino 1: 5′ ACGACCTCTTCAGAGTCCGACATGC3′ and morpholino 2: 5′TGGCTATCTAGCAGAACGCAAGGCA3′). In addition, a standard control (SC) morpholino was designed to mutated human β‐*globin* (5′CCTCTTACCTCAGTTACAATTTATA3′; GeneTools LLC, USA). Morpholinos were tagged with fluorescein to detect uptake by the embryo. A BLASTN search of each morpholino showed 100% homology of the morpholino nucleotides only to the gene of interest with E‐values close to zero. Untreated controls (UT) were also included, in which the embryos underwent the same treatment as the experimental and SC groups, except morpholino/pluronic gel mix was not applied. TNNT2‐MO treatment was conducted at stage HH11 (approximately 47–50 h after incubation; heart undergoing dextral rotation and the embryo has 13 somite pairs). Morpholino was resuspended in Hanks’ Balanced Salt Solution (HBSS) to give a final concentration of 15% F127 pluronic gel (BASF Corp., Germany) and either 250 or 500 μm morpholino was applied to embryos. The eggs were reincubated until they reached HH19 (75–80 h; unpigmented eyes, straight trunk and upper limbs extending across six somites). Animal work was completed within national (UK home office) and institutional regulations and ethical guidelines. Embryos were isolated at HH19 and morpholino uptake was confirmed by the fluorescein tag within the embryonic tissue using a SV11 stereomicroscope (Zeiss, Germany). Fluorescein positive embryos were collected along with UT controls. Embryos collected for the phenotypic and immunohistochemistry studies were placed in 4% paraformaldehyde (PFA), and embryos isolated for Western blot were snap‐frozen in liquid nitrogen and stored at −80 °C.

### Western blot

Individual hearts (TNNT2‐MO‐treated, SC and UT) were lysed in 60 μL 10 mm Tris/EDTA buffer containing protease inhibitors. 15 μL of 6×  loading buffer was added and samples were heated to 95 °C for 5 min. Samples were resolved by SDS‐PAGE in a 4–12% precast gel (Bio‐Rad, UK) along with Precision Plus Protein Dual Colour Standards (Bio‐Rad). Gels were transferred onto nitrocellulose membrane. Membranes were blocked in 10% bovine serum albumin (BSA) TBST for 1 h at room temperature (RT), followed by overnight incubation with CT3 anti‐mouse antibody (1 : 10 dilution; DSHB) at 4 °C. As a loading control, blots were incubated with 1 : 2000 rabbit polyclonal GAPDH (Abcam, UK) for 1 h at RT. Secondary antibodies were incubated for 1 h at 37 °C at 1 : 10 000 dilutions (IRDye® 800CW Goat anti‐rabbit and 680RD Goat anti‐mouse secondary antibodies; LI‐COR, USA). Antibody detection was completed using the Odyssey Infrared Imaging System (LI‐COR). All immunoblots contained TNNT2‐MO‐treated (otherwise called cTNT knockdown hearts), SC and UT samples, and the Western was carried out four times. Fluorescent intensities of the bands were gathered for statistical analysis.

### Phenotypic analysis

Embryos positive for morpholino uptake and UT controls were externally examined at the time of harvesting. The overall structure and size of the heart were observed as well as the embryo as a whole. Images were taken using Stemi SV11 stereomicroscope and camera (Zeiss). Embryos harvested and externally analysed were fixed at room temperature (RT) in 4% PFA in phosphate‐buffered saline (PBS) for 1.5 h, washed and stored in PBS overnight. Embryos were washed with H_2_O and dehydrated in 70–100% series of ethanol, cleared with xylene and embedded in paraffin wax in a transverse orientation. Each embryo was sectioned transversely (cranial to caudal) at 8 μm using a DSC1 microtome (Leica, Germany). Sections were dewaxed in xylene, rehydrated in a graded ethanol series, and stained with nuclear stain Mayer's haemalum (Fisher Scientific, UK). Internal features such as atrial septa, endocardial cushions, cardiac jelly and ventricular trabeculae were examined. Analysis was performed blind using a Nikon Eclipse microscope (Nikon Instruments Inc., UK).

### Histological and immunohistochemical analysis of morpholino‐treated hearts with diverticula

HH11/19 TNNT2‐MO‐treated hearts were isolated and processed for wax embedding as described above. Embryos with diverticula were serially sectioned at 6 μm sagittally, dewaxed and rehydrated in graded ethanol series and water, with neighbouring sections placed on adjacent slides to allow for the analysis of different stains. For the histological study, one set of sections were stained with extracellular matrix glycosaminoglycan Alcian Blue for 15 min at RT followed by Mayer's haemalum.

For the fluorescence immunohistochemistry, antigen retrieval was performed (microwaving for 15 min in 10 mm citric acid buffer pH 6.0) on a second set of sections, and blocked in 5% pre‐immune goat serum in 1% BSA/PBS for 30 min at RT. Primary antibody, A4.1025 (a marker for sarcomeric myosin heavy chain; 1 : 10; DSHB, USA) was diluted in 1% BSA/PBS and incubated for 1 h at RT. Sections were incubated with secondary antibody Cy2 (1 : 100; A4.1025; Jackson ImmunoResearch Inc., USA) for 30 min at RT. Nuclei were stained with DAPI (4’,6‐diamidino‐2‐phenylindole; 1 : 1000; Sigma) and imaging was carried out on a LSM5 Exciter confocal microscope (Zeiss).

For the Masson's Trichrome stain, a third set of sections from the TNNT2‐MO‐treated embryonic hearts with diverticula were mordanted in Bouin's fixative for 1 h at 56 °C. They were then stained with nuclei stain Weigert's iron haematoxylin working solution for 8 min, Biebrich scarlet‐acid fuchsin for muscle stain for 5 min, phosphotungstic‐phosphomolybdic acid for 12 min, and aniline blue solution for collagen stain for 5 min. Sections were differentiated in 1% acetic acid followed by dehydration. Images were taken using an Axioplan microscope (Zeiss).

### Stereological systematic random sampling of morpholino treated embryos

Systematic random sampling was used to quantify cardiac tissue proportions (HH11/19). Three groups were analysed: untreated controls (UT; *n* = 3), standard controls (SC; *n* = 4) and TNNT2‐MO‐treated hearts (*n* = 9). A 96‐point grid was placed over every third section throughout the heart, and the tissue region and type on each point were identified (7900 points counted). Tissue regions consisted of the atrium, ventricle and OFT, with the boundaries between the compartments defined by changes to myocardial and cardiac jelly thickness. The tissue types counted included myocardial wall, extracellular matrix and lumen in each of the regions mentioned. The average tissue proportions were calculated and tested for statistical significance.

### Isolation of cell micromass from embryonic chick hearts and morpholino treatment

White fertile chicken eggs were incubated for 5 days (HH26; three digits in lower limb; elbows defined in the upper limb, with longer, rounded limb buds) as described above. Eggs were washed with trigene, swabbed with IMS and placed in a clean and sterilized class II Laminar flow hood. The embryos were isolated in 1 : 1 horse serum/HBSS on ice. Once explanted, the hearts were washed in HBSS and placed in trypsin/EDTA (0.05%) at 37 °C, 5% CO_2_ for 20 min. The hearts were triturated until homogenized and 8 mL of culture media (10% heat inactivated fetal bovine serum, 2 mm L‐glutamine and 50 units mL^−1^ penicillin/50 μg mL^−1^ streptomycin was added to 500 mL Dulbecco's modified Eagle's medium and nutrient mixture F‐12 HAM) was added. The suspension was centrifuged and the pellet resuspended in 2 mL of warm culture media. The cell suspension was seeded in a 24‐well plate containing glass coverslips at a cell density of 6 × 10^5^ cells mL^−1^. The cells were left in the CO_2_ incubator for 2 h to allow attachment to the coverslips before adding 500 μL of warm culture medium. TNNT2‐MO was pre‐heated to 65 °C for 5 min. 3 μL of Endo‐Porter (GeneTools) was added to 250 μL of pre‐warmed culture medium and 10 mm morpholino. SC morpholino and no treatment were used as controls. The morpholino mix was added to 250 μL of culture medium in each well. Cells were left for 48 h in a CO_2_ incubator.

### Immunofluorescent staining and analysis of TNNT2 treatment in culture

After 48 h, the culture media was removed, cells were fixed for 10 min in 4% PFA at RT and permeabilized with 0.2% Triton‐X 100 in PBS. CH1 mouse monoclonal antibody (anti‐tropomyosin antibody; DSHB, USA) was prepared at a 1 : 50 dilution in 1% BSA in PBS and incubated with the cells overnight at 4 °C. The cells were rinsed with PBS and goat anti‐mouse secondary antibody, Alexa Fluor 596 (1 : 1500; Thermo Fisher Scientific Inc., UK) was incubated with the cells for 40 min at RT. Cells were mounted on glass slides (GeneTex, USA) and stored at 4 °C. Cells were visualized using the DMIRE2 inverted microscope (Leica) and cells positive for CH1 antibody were considered cardiomyocytes. Cardiomyocytes positive for morpholino uptake were included in the study. The assembly of the sarcomere was divided into four stages, according to the degree of myofibrillogenesis. Over 530 cells were analyzed for this study.

### Quantitative PCR

HH19 hearts were dissected from TNNT2‐MO‐treated and control embryos. Total RNA of individual hearts was extracted using Norgen Total RNA Purification Kit (Norgen Biotek) according to the manufacturer's protocol. RNA was finally eluted from the columns with 50 μL of RNase‐free water. The concentration and purity of the eluted RNA samples were determined using the NanoDrop 2000 c UV/IV spectrophotometer at 260 nm absorbance (Thermo Fisher Scientific). RNA samples were stored at −80 °C. Synthesis of cDNA template was performed using 1 μg RNA and random primers with SuperScript II Reverse Transcriptase (Invitrogen, UK). Each cDNA sample was diluted 4.5×  with RNase‐free water before the determination of the dynamic range of sample concentrations for the qPCR experiment. From the standard curve obtained, a concentration was chosen for relative quantitation that fell in the middle of the slope. Efficiencies of all gene reactions were between 93 and 108%, with r^2^‐values > 0.998. qPCR was performed using the Applied Biosystems 7500 Fast Real‐time PCR system under the following thermal cycling conditions: a single heat‐denaturing step at 95 °C for 10 min, followed by 40 cycles of annealing and extension at 95 °C for 20 s and 62 °C for 1 min. A melt curve analysis was performed from 50 to 95 °C, with no off‐target products detected. All samples were run in triplicate within each PCR experiment. Each 20 μL PCR reaction mixture consisted of 10 μL of iTaq™ universal SYBR^®^ Green supermix (1×), 0.5 μL of each forward and reverse primer (250 nm) for all genes and 2 μL of diluted cDNA template (1 in 11 dilution for *TBX3* and 1 in 6 dilution for *NOS3*). Relative gene expression was quantified with *GAPDH* as the reference gene. The following primer sequences, spanning an intron, were used for qPCR: *NOS3* (GenBank accession number XM_003640632.2), 5′ACCTACCAGAGCGAGAACGG3′ and 5′CTGCCAGAAGCTGCGGAATG3′ (198 bp); *TBX3* (NM_001270878.1), 5′AGTGCACCTGGAAGCCAAAGA3′ and 5′GTCGTCAGCCGCCACAATATC3′ (169 bp) and *GAPDH* (NM_204305.1), 5′AGACGGTGGATGGCCCCTCT3′ and 5′ACGGCAGGTCAGGTCAACAACA3′ (263 bp).

### Statistics

Levene's test for equality of variances was completed, followed by a *t*‐test for equality of means on SPSS V21.0 (SPSS Inc., USA), *P *<* *0.05 was considered as significant. Where appropriate, the mean ± standard error of the mean (SEM) was calculated.

## Results

### Differential expression of *TNNT2* isoforms in the chick heart

**Figure 1:**
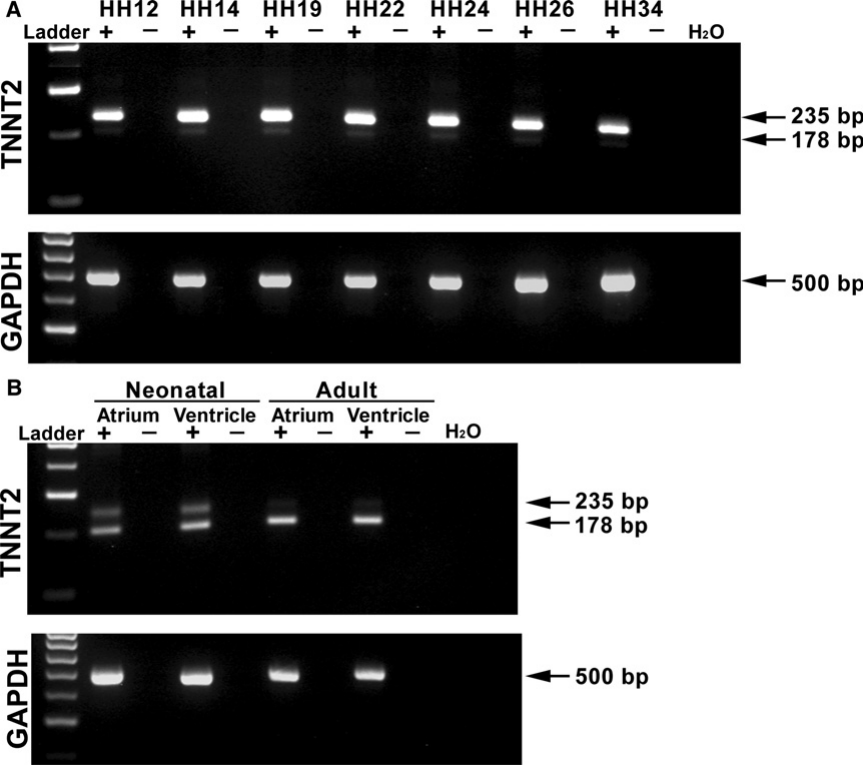
mRNA expression of *TNNT2* in the embryonic, neonatal and adult chick heart. PCR products obtained using a primer pair designed around the exon 5, which is alternately spliced in *TNNT2*. (A) Expression of a *TNNT2* isoform in the embryonic chick heart (HH12–34; 235 bp). A smaller band of 178 bp can also be seen faintly expressed. *GAPDH* was used as a loading control (500 bp). (B) Two bands can be clearly seen in both the atrium and ventricle of the neonatal heart. In the adult atrium and ventricle, however, the lower band has a stronger signal than the upper 235 bp band. *GAPDH* was used as a loading control. + indicates RT; −, noRT; bp, base pairs; H_2_O, PCR control.

To examine the differential expression of *TNNT2* in the chick heart, a gene‐specific primer pair was designed around exon 5 (present in embryonic forms of *TNNT2* but absent in the adult). cDNA was obtained from HH12, HH14, HH19, HH22, HH24, HH26 and HH34 hearts and from neonatal and adult atrium and ventricles. During embryonic stages of development, an intense 235 bp band is present, with a faint expression of a 178 bp band below (Fig. 1A). In the neonatal and adult atrium and ventricle, there appears to be a shift in expression, as expected, with the upper band becoming less intense and the lower band intensely expressed at 178 bp (Fig. 1B). The upper 235‐bp band contains exon 5, whereas the lower band is formed by alternate splicing omitting exon 5 from the RNA sequence. *Glyceraldehyde 3‐phosphate dehydrogenase* (*GAPDH*) was utilized as a loading control (Fig. 1A,B).

### Embryo grouping, survival and TNNT2‐MO uptake

**Figure 2:**
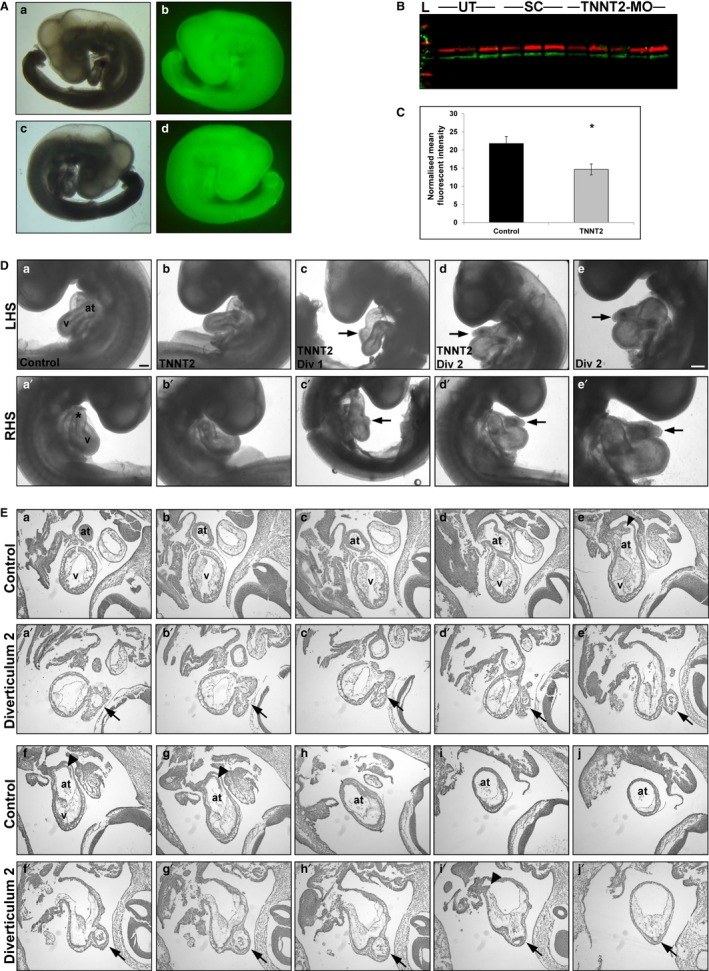
TNNT2‐MO treatment results in a knockdown of cTNT and external analysis reveals diverticula protruding from the heart wall. (A) Embryos were treated at HH11 with a TNNT2 or standard control fluorescein‐tagged morpholino; only embryos showing a strong fluorescent signal were harvested (at HH19). (b,d) Fluorescent embryo in comparison to brightfield a,c; both left‐ (a,b) and right‐hand side (c,d) shown. (B,C) Western blot of individual hearts treated with either TNNT2‐MO, standard control morpholino or untreated at HH11 results in a significant decrease of cTNT at HH19 (*P *=* *0.016). On (B) the lanes 1–3 are untreated, 4–6 have had standard morpholino applied, and 7–11 are TNNT2‐MO‐treated. (D) External analysis of embryos treated with TNNT2‐MO reveals normal heart development in the majority of embryos (b and b') when compared with the controls (a and a'). However, two embryos presented with diverticula on the heart wall (c and c', d and d'). Diverticulum 2 is shown at higher magnification (e and e'). (E) Serial sectioning through a control (a‐j) and embryo with diverticulum 2 (a'‐j'). Three opening are present between the ventricular chamber into the diverticulum (b', f'and i'). Arrowheads denote the normal atrial septa in controls (f and G), which is reduced in diverticulum 2 heart (i'). *, outflow tract; arrowhead, atrial septum; arrows, diverticulum; at, atrium; LHS, left‐hand side of embryo; RHS, right‐hand side of embryo; v, ventricle; Scale bars: 100 μm.

Two concentrations of TNNT2‐MO, 250 and 500 μm, were initially used to determine the optimal concentration. HH11/19 represents morpholino treatment at HH11, and harvesting at HH19. For the 250 μm study, the survival rates for untreated (UT) chick embryos (*n* = 22), standard control (SC) group (*n* = 30) and TNNT2‐treated (*n* = 43) embryos were 90.9, 94.1 and 91.5%, respectively (*P *>* *0.298). Survival rates for the 500‐μm study for UT (*n* = 52), SC (*n* = 56) and TNNT2‐MO‐treated group (*n* = 106) were 96.1, 91.7 and 92.9%, respectively (*P *>* *0.275). Surviving embryos were examined for ‘morpholino uptake’ determined by the degree of fluorescence (Fig. 2A). In the 250‐μm study, 43.8% of the SC and 58.1% of the TNNT2‐MO‐treated embryos were positive for morpholino uptake (*P *=* *0.448). For the 500‐μm study, 63.6% of the SC and 73.6% of the TNNT2‐MO‐treated embryos were positive (*P *=* *0.446).

### TNNT2‐MO treatment results in decreased cTNT in the heart

Western blot analysis was used to quantify the degree of protein ‘knockdown’ in individual hearts treated with TNNT2 ATG start‐site morpholino. CT3 antibody detecting cTNT was normalized using GAPDH. No significant difference between the UT and SC groups (*n* = 3) was seen (*P *=* *0.233) and these two groups were therefore pooled into one control group. When the control and TNNT2‐treated hearts (*n* = 5) were compared, TNNT2 was significantly decreased in the TNNT2‐treated hearts and knockdown was achieved (*P *=* *0.016; Fig. 2B,C).

### TNNT2‐MO treatment results in the development of a diverticulum in the ventricular wall

Once embryos were confirmed positive for morpholino uptake, embryos were phenotypically examined. External features such as looping; atrial and ventricular positioning and size; length and shape of the outflow tract (OFT) were studied. No external phenotype was observed for the 250‐μm study for either the controls (*n* = 34) or TNNT2‐MO‐treated (*n* = 43) embryos (see Table 1 for a summary of TNNT2‐MO experiments). For the 500‐μm study, all controls (*n* = 84) and the majority of TNNT2‐MO‐treated embryos (*n* = 67) appeared to have a normal external phenotype (Fig. 2Da,b'). However, two of the TNNT2‐MO‐treated hearts presented with a diverticulum on the heart wall. Both diverticula were situated at the anterior part of the primitive ventricle, with one present inferior to the OFT (diverticulum 1; Fig. 2Dc,c') and one inferior to the atrium (diverticulum 2; Fig. 2Dd‐e').

**Table 1:** Summary of phenotypic analysis after treatment with TNNT2 morpholino

Concentrationof morpholino	Embryo type^a^	External heart shape			Internal heart structures				Total internal
					Atrial septum		Trabeculation		
		Diverticulum present	Normal	Total external	Reduced	Normal	Reduced	Normal	
500 μm	Control	–	84	84	1	37	–	38	38
	TNNT2	2^b^	65	67	23	28	6	45	51
250 μm	Control	–	34	34	–	15	–	15	15
	TNNT2	–	43	43	–	19	–	19	19

Assessment of heart shape based on qualitative analysis of gross morphological features.

Qualitative assessment of atrial septa and degree of trabeculation obtained from serial histological sections.

TNNT2 denotes embryos treated with the TNNT2 ATG start site morpholinos.

a Control embryos include standard and untreated controls.

b Both embryos with diverticula had reduced atrial septal size.

### Normal morphology and histology of the novel diverticulum on TNNT2‐MO‐treated embryonic hearts

To further investigate the ventricular diverticula, both hearts were sectioned serially, and consecutive sections collected on adjacent slides to allow for analyses with different markers. A set of these sections were Alcian blue‐ and haemalum‐stained. The first diverticulum appeared as a mild protuberance of the myocardium and endocardium located just inferior to the OFT, on the anterior part of the right side of the primitive ventricle. The second diverticulum appeared to have a more complex structure compared to the first diverticulum. Fig. 2E compares a control heart with a normal gross morphology with the second diverticulum, both sectioned sagitally. The control heart shows the structure of a normal heart progressing through the ventricle and atrium (Fig. 2Ea‐j). In the images for the second diverticulum, we first saw the appearance of the diverticulum on the anterior part of the left side of the primitive ventricle inferior to the primitive atrium (arrow in Fig. 2Ea'). As the sections progress through the ventricular chamber, a communication appears between the ventricle and the diverticulum (Fig. 2Eb'). This communication quickly closes (Fig. 2Ec'‐e'). Towards the end of the ventricular chamber, as the atrioventricular canal and atrial chamber appears, another communication can be seen (Fig. 2Ef'). Again, the communication closes (Fig. 2Eg'‐h') until the diverticulum reopens for the final time in the atrial chamber (Fig. 2Ei'‐j').

**Figure 3:**
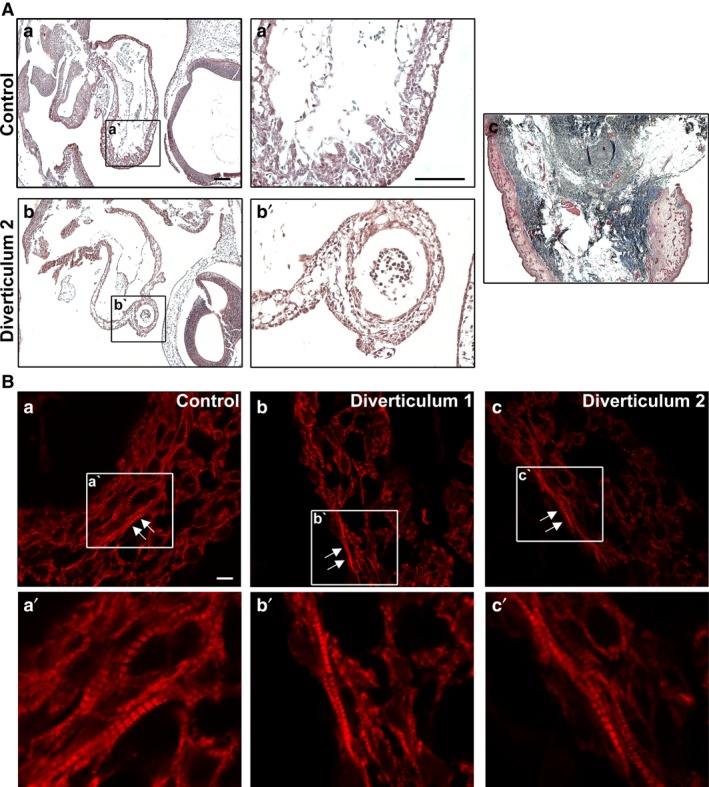
Histological analysis of the diverticula in the TNNT2‐MO‐treated embryos. (A) Masson's Trichrome staining was used to detect collagen that may have been deposited due to fibrosis of the heart. When compared with a control heart (a and a'), the diverticula did not appear to show a noticeable change in collagen deposition that would be indicated by the black/blue staining (b and b'). Chick skin was used as a control for the Trichrome stain (c). Scale bars: (a,a')  300 μm. (B) An anti‐myosin heavy chain antibody was used to detect cardiac muscle in the heart. When compared with the control (a), diverticulum 1 and 2 had normal cardiac muscle appearance, with the presence of mature myofibrils in the myocardial wall (arrows; b and c). a', b' and c' show sarcomeres at higher magnification from images a, b and c, respectively. Scale bar: 80 μm.

To investigate whether the diverticula were of the muscular or fibrous type, Masson's trichrome staining was also completed on a third set of neighbouring sections to detect excess collagen deposits within the diverticula. This staining technique revealed no abnormal collagen deposits within the diverticula indicating a non‐fibrous phenotype (Fig. 3Ab,b') in comparison with control heart (Fig. 3Aa,a'). To complement this data, anti‐myosin antibody staining was performed on a third set of neighbouring sections to detect muscle fibres within the hearts with diverticula. When compared with a control heart (Fig. 3Ba,a'), the muscle fibres appeared normal in the myocardium of both diverticula (Fig. 3Bb‐c').

### TNNT2‐MO treatment results in abnormal atrial septa formation

**Figure 4:**
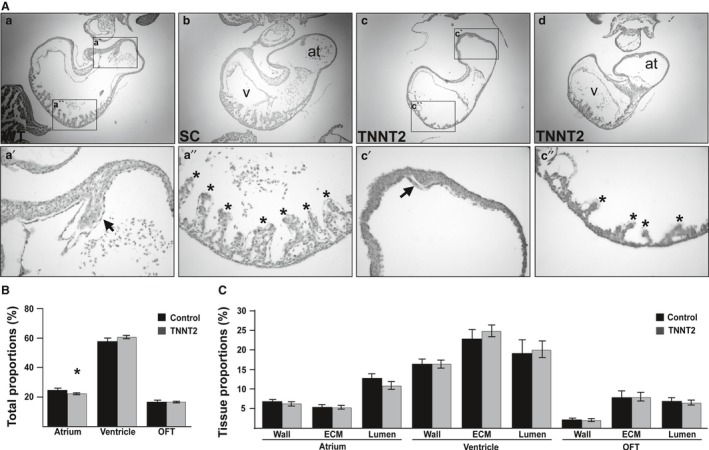
TNNT2‐treated embryos undergo abnormal atrial septation and trabecular formation, and the atrial chamber is reduced in size. (A) Internal analysis of the UT and SC hearts revealed normal structures (a, b), with initiation and growth of an atrial septum (a') and trabeculae (a''). In contrast, although TNNT2‐treated hearts displayed atrial septation initiation, the atrial septum in these hearts appeared small in size (c,c',d). In the ventricles, the trabeculae of a subset of the atrial phenotypic hearts were reduced in size and numbers (c,c''). (B) Upon stereological analysis, the atrial contributions to the total heart size was decreased in TNNT2‐treated hearts; this decrease appeared small but was statistically significant (*P *=* *0.045), whereas the OFT and ventricular contributions to heart size remained the same (*P *>* *0.053). (C) No difference in tissue proportions contributing to each region of the heart was seen upon stereological analysis (*P *>* *0.120). *, trabeculae; arrow, septum; at, atrium; v, ventricle. Scale bars: 100 μm.

HH11/19 embryos were serially sectioned in a transverse orientation to investigate the development of the atrial septum from the dorsocranial wall of the common atrium. For the 250‐μm study, normal atrial septation was present in all control (*n* = 15) and TNNT2‐MO‐treated groups (*n* = 19), with all septa appearing to have a normal size (Table 1). In the 500‐μm study, the atrial septa appear to develop normally in all but one of the controls (*n* = 37; Table 1; Fig. 4Aa,b,a'). In the TNNT2‐MO‐treated embryos, atrial septation was induced in all hearts. However, in 45% of these TNNT2‐MO hearts, the septum appeared small and resembled a knuckle‐shaped outgrowth from the atrial myocardium (*n* = 23/51; Table 1; Fig. 4Ac,d,c'). This includes the two hearts with the diverticula (see arrowhead in Fig. 2Ei'; control Fig. 2Ef,g).

### Trabeculation of the ventricular myocardium is affected in a subset of the hearts with an abnormal atrial septum

Normal development of the ventricular myocardium was also assessed in the TNNT2‐MO‐treated embryos. Trabeculae, finger‐like muscular projections when sectioned transversely, appear normal in the UT and SC groups in both the 500‐μm and 250‐μm studies (*n* = 15 and *n* = 38, respectively; Table 1; Fig. 4Aa,b,a'') and appeared normal for all of the 250‐μm TNNT2‐MO‐treated group (*n* = 19). In the 500‐μm study, almost all embryos appear to have normal trabeculation (Fig. 4Ac); however, a small proportion (11.8%) of the hearts appeared to have a reduced number and size of trabeculae (6/51; Fig. 4Ac,c''). Interestingly, all the hearts displaying reduced ventricular trabeculae also had a reduced atrial septum.

### Stereological analysis reveals reduced atrial chamber size but normal tissue proportions throughout the heart

Stereological analysis was completed on TNNT2‐MO‐treated embryos to obtain information regarding tissue proportions compared with control hearts. For the control hearts (UT *n* = 3; SC *n* = 4) 3978 points were counted, and for the TNNT2‐MO‐treated hearts, 3922 points were counted (*n* = 9). A percentage proportion of the atrium, ventricle and OFT was first achieved by dividing the total number of counts per region by the total number of counts for that heart (Fig. 4B). No significant difference was seen between the UT and SC groups (*P *>* *0.598) and the two groups were therefore pooled. Upon comparing the total proportion the atrium contributed to the heart size, the atrium in the TNNT2‐MO‐treated hearts was smaller than in the controls (22.32 ± 0.53 and 24.85 ± 1.12%, respectively; 0.045 μm ; Fig. 4B). No significant difference in proportions was seen in the OFT and ventricle between the control and knockdown hearts (Fig. 4B; *P *>* *0.053). The percentage of lumen, myocardium and ECM within each heart region (OFT, ventricle and atrium) was also analyzed using this stereological method. As no significant difference was seen between the UT and SC groups, they were pooled (*P *>* *0.091). No differences were seen in any of the heart regions between the controls and the TNNT2‐MO‐treated hearts (Fig. 4C; *P *>* *0.120).

### TNNT2‐MO treatment in culture does not result in aberrant sarcomere assembly

**Figure 5:**
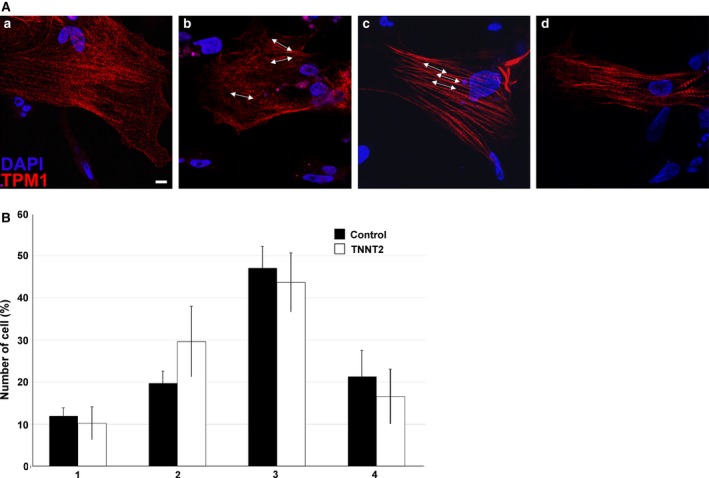
TNNT2‐MO treatment does not result in aberrant myofibrillogenesis. (A) An example of non‐treated cardiomyocytes in culture. Sarcomere assembly was categorized into four types: type 1 is the immature myofibril which is assembling at the periphery of the cell and no fibril structures can be seen (a); type 2 is when fibres are present but in a disorganized fashion (b); type 3 has organized but still thin fibrils (c); type 4 has fully developed thick fibrils running from one end of the cell to the other (d). Arrows indicate the direction of the myofilaments. Scale bar: 80 μm. (B) TNNT2‐MO treatment does not appear to affect sarcomere assembly and maturity (*P *>* *0.180).

The effect of TNNT2‐MO treatment on sarcomere assembly was investigated using cardiac cell micromass. Over 500 cardiomyocytes that showed uptake of the fluorescein‐tagged morpholino were analysed in four independent studies. Using an anti‐TPM1 antibody to detect the sarcomere, we classified each cardiomyocyte into one of four stages depending on its sarcomeric maturity. Stage 1 consisted of cells with positive staining around the periphery of the cell but no sarcomeric structures can be clearly seen, especially around the nucleus (Fig. 5Aa). Stage 2 cardiomyocytes contain assembled sarcomeres; however, they appear thin and disorganized (Fig. 5Ab). Stage 3 cardiomyocytes present with organized sarcomeres that appear thin but are organized and run parallel to one‐another (Fig. 5Ac). Finally, stage 4 cardiomyocytes contain mature cardiomyocytes with thick bands running through the cell (Fig. 5 Ad). As no significant difference was seen between UT and SC‐treated cells these data were pooled (*P* > 0.582). When the control group was compared with the TNNT2‐MO‐treated group, no significant difference was seen in sarcomere assembly in any of the groups (*P* > 0.180).

### TNNT2‐MO treatment alters the expression of *NOS3*, a shear stress marker, within the embryonic heart

**Figure 6:**
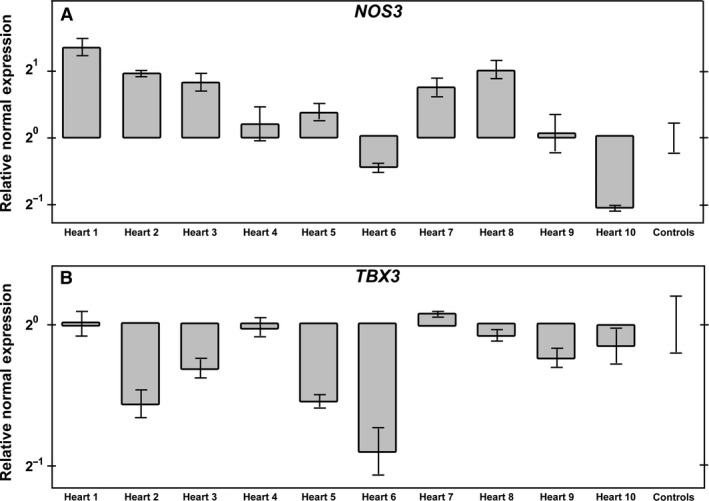
TNNT2‐MO treatment alters the expression of *NOS3* and *TBX3* in the developing heart. (A) *NOS3* expression is increased in hearts 1, 2 and 8 when compared with the control. In heart 10, the expression of *NOS3* is decreased. The expression in hearts 3, 4, 5, 6, 7 and 9 remain comparable to that in controls. (B) *TBX3* expression is decreased in hearts 2, 5 and 6 when compared with the control. The expression of TBX3 in hearts 1, 4, 7, 8, 9 and 10 remains comparable to that in the control.

During heart development, normal haemodynamics is important for normal development, and blood flow correlates with the shear stress placed upon the heart. *NOS3* is a shear stress‐responsive gene, and known to be essential for atrial septation (Feng et al. 2002; Groenendijk et al. 2005; Liu & Feng, 2012). Therefore, to investigate the potential cause of the abnormal atrial septum and reduced trabeculae in the TNNT2‐MO‐treated hearts, *NOS3* expression was quantitatively analysed. qPCR was conducted using 10 individual TNNT2‐MO‐treated embryonic hearts to evaluate a trend in expression after morpholino treatment. As no significant difference in expression was identified between the controls, they were pooled (*n* = 3). Of the 10 TNNT2‐MO‐treated hearts, the expression of *NOS3* was elevated in three (30%; hearts 1, 2 and 8; Fig. 6A). Within these hearts, there was a 2.57‐, 1.96‐ and 2.02‐fold increase of *NOS3*, respectively. Conversely, heart 10 had a 2.08‐fold decrease in *NOS3* expression, whereas the remaining six hearts appeared to have no change in *NOS3* expression compared with controls (60%; hearts 3, 4, 5, 6, 7 and 9).

### TNNT2‐MO treatment during heart development alters *TBX3* expression, a marker of the developing conduction system

Previous studies have associated structural proteins with abnormal electrical activation of the heart and with sinus node dysfunction (Holm et al. 2011; Rutland et al. 2011). Using *TBX3* as a marker for the maturing conduction system, quantification of *TBX3* expression was analysed in individual TNNT2‐MO‐treated hearts. Again, no significant difference was seen between three SC morpholino‐treated hearts and they were pooled as a control. Seven of the 10 TNNT2‐MO hearts appeared to have normal expression when compared with the control (hearts 1, 3, 4, 7, 8, 9 and 10; Fig. 6B). Interestingly, 30% of the hearts had reduced expression of *TBX3* (hearts 2, 5 and 6, showing a 1.54‐, 1.5‐ and 1.88‐fold down regulation, respectively; Fig. 6B).

## Discussion

cTNT is a vital sarcomeric protein expressed in the developing chick heart. As shown in this study, *TNNT2* isoforms are expressed as early as HH12 in the developing chick heart and are present throughout development. Using RT‐PCR and knowledge of *TNNT2* alternate splicing, we have shown that the fetal isoforms are predominantly expressed in the developing embryonic heart (Fig. 1). In the neonatal heart, there appears to be an isoformal shift occurring, where both fetal and adult isoforms are detected. In the adult heart, the fetal isoform appears decreased with the predominant expression of the adult isoform (which does not contain exon 5).

Targeted manipulation of cTNT *in ovo* using morpholinos results in the development of a diverticulum from the wall of the primitive ventricle. According to the literature, diverticula are early embryological defects that usually arise from the left ventricle apex as a congenital left ventricular diverticulum, although many cases of right ventricular and both atrial diverticula have been reported (Binder et al. 2000; Caldaroni et al. 2014; Ohlow et al. 2015). Ventricular diverticula can be either muscular or fibrous, and they may or may not be associated with other CHDs, such as ASDs, ventricular septal defects, persistent ductus arteriosus, coarctation of aorta and conduction defects (Villegas Garcia et al. 2001; Pitol et al. 2005; Di Sessa et al. 2006; Ohlow, 2006; Ohlow et al. 2015). Although ventricular diverticula can remain asymptomatic, congestive heart failure, systemic embolism and sudden cardiac death may occur. Congenital ventricular diverticula have also on occasion been associated with hypertrophic cardiomyopathy (Maron et al. 1996). However, our embryonic hearts appeared to have a normal size and did not appear enlarged, although mutations in *TNNT2* have been associated with cardiomyopathy in the literature (Watkins et al. 1995).

One of the diverticula observed in our study (diverticulum 2; Fig. 2Dd,d') had similar morphology observed to the diverticulum seen in humans. Based on the phenotypic analysis, the ‘outpouching’ was connected to the primitive ventricle, atrioventricular canal and atrium through narrow communications (Fig. 2E), and had systolic contractility that was synchronous with the ventricle. Diverticulum 1 in this study had a different morphology in that it did not appear as an extra chamber and appeared more similar to a mild protuberance from the myocardial wall (Fig. 2Dc,c'). In addition, Masson's trichrome stain did not reveal fibrosis in the myocardium of the diverticula. Furthermore, an anti‐myosin antibody revealed normal sarcomeric structures in the myocardium, indicating that the diverticula presented here are of the muscular type. In the literature, the muscular type of cardiac diverticulum is often localized on the inferior or anterior walls of the left ventricle at the apex (Ohlow et al. 2015). In our case, we found our diverticulum 1 and diverticulum 2 were localized on the anterior part of the primitive ventricle inferior to the OFT and primitive atrium, respectively but not at the apex. Also, it has been proposed that the cardiac diverticulum is a developmental defect that potentially forms as a result of the structural muscle weakness of the heart (Lowe et al. 1959; Ohlow, 2006). Here, we postulate that the reduction of cTnT protein leads to muscular weakness and thus formation of the diverticulum. To our knowledge, this is the first report linking knockdown of sarcomeric proteins to diverticula. This diverticula phenotype was never present in any of the controls or, in fact, in any other morpholino experiments conducted by us or other laboratories targeting different sarcomeric genes (Ching et al. 2005; Matsson et al. 2008; Rutland et al. 2009, 2011). Although the phenotype was infrequent, it is believed that left ventricular diverticulum occur in only 0.04% of the general population. Interestingly, the literature reports the co‐existence of muscular congenital cardiac diverticula with ASDs (Walton‐Shirley et al. 1992; Shahmohammadi et al. 2008; Ohlow et al. 2015); in this study, in both of the hearts with a diverticulum, a reduced atrial septum was also found.

Stereological investigation did reveal that upon TNNT2‐MO treatment, the proportion of the atrium relative to the remaining heart was smaller than that of the control hearts (Fig. 4B). It is unknown whether this reduction in size is due to underdevelopment of the chamber itself, or caused by some haemodynamic changes through the atrial chamber. In addition, TNNT2‐MO treatment at the early stages of cardiogenesis results in abnormal septal growth in 45% of treated hearts. The septa appeared as knuckle‐shaped outgrowths from the dorsocranial atrial wall (Fig. 4A). This was also true for the atrial septum in the hearts with diverticula (Fig. 2D). The ventricular region of the cTNT knockdown hearts, in general, appeared phenotypically normal. However, a subset of hearts had reduced trabeculation in the ventricle (11.8%; Fig. 4A). During embryonic development, normal haemodynamics is critical for the development of structures within the heart and associated vasculature (Hogers et al. 1999; Hove et al. 2003; Granados‐Riveron & Brook, 2012). Importantly, shear stress induces *NOS3* expression during chick cardiogenesis (Groenendijk et al. 2005). *NOS3* is expressed in the atrial septum and ventricular trabeculae (Groenendijk et al. 2005), with chick atrial septation initiated at approximately HH14. In the *Nos3* ^*–/–*^ mouse, there was a high incidence of septal defects (64% ASD; 11% ventricular septal defects) (Feng et al. 2002; Liu & Feng, 2012), most likely due to an increase in apoptosis and reduction in proliferation (Lepic et al. 2006). Further, *Nos3* is known to interact with cardiac transcription factor *Tbx5*, with *Tbx5* expressed in endocardial cells that will contribute to the atrial septum (Nadeau et al. 2010). From the analysis of genetic mutants and *in vitro* studies, *Nos3* is predicted to be a genetic modifier of *Tbx5* in atrial septa formation (Nadeau et al. 2010). Therefore, we looked at the expression of *NOS3* in individual TNNT2‐MO‐treated embryonic hearts to see if any alterations in shear stress may be occurring. In our study, variable expression of *NOS3* was seen in TNNT2‐MO‐treated hearts by qPCR, with three hearts showing increased and one heart a reduction in expression compared with controls, suggesting the hearts are responding differently to the altered blood flow. Future studies on apoptosis and proliferation in the TNNT2‐MO‐treated hearts may elucidate whether these processes contribute to the abnormal structural phenotypes observed.

In the chick, pacemaker‐like activity has been found in a small population of progenitor cells at HH8 (Bressan et al. 2013). The transcription factor *TBX3* is a marker of the developing conduction system, with expression seen in the sinoatrial region at E8.5/9 in the murine heart (Binder et al. 2000; Hoogaars et al. 2004; Baruteau et al. 2015; van Weerd & Christoffels, 2016). Analysis of a transgenic mutant at E9.5 in the mouse showed that overexpression of *Tbx3* inhibited myocardial differentiation by suppression of chamber genes, such as *Nappa* and *Cx40* (Mommersteeg et al. 2007). The *Nppa* promoter is known to be activated by *Tbx5,* an interaction which can be prevented by *Tbx3* (Hoogaars et al. 2004); Tbx5 is also known to be important in atrial septation (Li et al. 1997). By inhibiting myocardial genes, *TBX3* permits the differentiation of the sinoatrial node, the AV bundles and bundle branches (Hoogaars et al. 2007; Mommersteeg et al. 2007; Bakker et al. 2008). Importantly, *Tbx3* appears to be sensitive to dosage with variable levels of under‐expression as well as ectopic expression in the mouse leading to a range of conduction anomalies, and hence has been proposed as a candidate gene for arrhythmias in humans (Hoogaars et al. 2007; Frank et al. 2012). In this study, qPCR of individual TNNT2‐MO‐treated hearts revealed a decrease in *TBX3* expression in three of the 10 hearts examined, which could lead to the aberrant formation of the early conduction system. Structural proteins have previously been associated with abnormal electrical activation of the heart and with sick sinus syndrome (Holm et al. 2011; Rutland et al. 2011). Further, conduction defects have accompanied a range of structural defects, including ASDs and congenital cardiac diverticula (Ohlow, 2006; Baruteau et al. 2015).

Although the *Tnnt2* ^–/–^ mouse was not viable due to the lack of sarcomeric organization and contractility (Ahmad et al. 2008), we found that TNNT2‐MO treatment did not affect sarcomere assembly. In the zebrafish mutant, Z‐bodies did form; however, pre‐myofibrils were not produced (Huang et al. 2009). In both the mouse and zebrafish models, targeted deletion of cTnT occurs prior to its expression in the developing embryo. In contrast, with the chick, morpholino treatment is completed at HH11, when the heart tube is present and there is already expression of sarcomeric proteins, including cTNT. Therefore, a protein pool of cTNT is already available to the embryo after knockdown using the morpholino. By allowing the initial heart tube to form prior to morpholino treatment, developmental processes such as atrial septation and maturation of the ventricular chamber can be studied in this model system, which was not possible in the Tnnt^*–/–*^ mouse, due to embryonic lethality at E10.5. In recent years, the specificity of morpholino oligonucleotides has come under scrutiny (Kok et al. 2015). However, many advantages remain for the use of morpholinos and are still useful entities when comprehensive controls are used (Blum et al. 2015).

To our knowledge, this is the first study associating *TNNT2* to abnormal atrial septal growth, reduced ventricular trabeculation and ventricular diverticula. The altered expression of *TBX3* and *NOS3* suggests that a reduction in cTnT in the developing heart affects important molecular signals required for normal development, and in turn results in the abnormal gross anatomical phenotypes observed in this study. Further investigations into haemodynamic changes and/or altered contractility within these hearts would be necessary to provide insights into the effects that reduced cTnT has on these processes. Furthermore, studies need to be performed on the *TNNT2* gene to find a link between *TNNT2* and CHDs in humans.
